# ω-Conotoxin GVIA Mimetics that Bind and Inhibit Neuronal Ca_v_2.2 Ion Channels

**DOI:** 10.3390/md10102349

**Published:** 2012-10-22

**Authors:** Charlotte Elisabet Tranberg, Aijun Yang, Irina Vette, Jeffrey R. McArthur, Jonathan B. Baell, Richard J. Lewis, Kellie L. Tuck, Peter J. Duggan

**Affiliations:** 1 CSIRO Materials Science and Engineering, Bag 10, Clayton South, Victoria 3169, Australia; Email: l.tranberg@deakin.edu.au; 2 Institute for Molecular Bioscience, The University of Queensland, St Lucia, QLD 4072, Australia; Email: aijun.yang@csiro.au (A.Y.); i.vetter@imb.uq.edu.au (I.V.); r.lewis@imb.uq.edu.au (R.J.L.); 3 Health Innovations Research Institute, RMIT University, Melbourne, Victoria 3083, Australia; Email: jeffrey.mcarthur@rmit.edu.au; 4 Medicinal Chemistry and Drug Action, Monash Institute of Pharmaceutical Sciences, Monash University, 381 Royal Parade, Parkville, Victoria 3052, Australia; Email: Jonathan.Baell@monash.edu; 5 School of Chemistry, Monash University, Clayton, Victoria 3800, Australia

**Keywords:** Ca_v_2.2, conotoxin, peptidomimetics, radioligand binding, Ca^2+^ fluorescence assay, patch clamp electrophysiology

## Abstract

The neuronal voltage-gated N-type calcium channel (Ca_v_2.2) is a validated target for the treatment of neuropathic pain. A small library of anthranilamide-derived ω-Conotoxin GVIA mimetics bearing the diphenylmethylpiperazine moiety were prepared and tested using three experimental measures of calcium channel blockade. These consisted of a ^125^I-ω-conotoxin GVIA displacement assay, a fluorescence-based calcium response assay with SH-SY5Y neuroblastoma cells, and a whole-cell patch clamp electrophysiology assay with HEK293 cells stably expressing human Ca_v_2.2 channels. A subset of compounds were active in all three assays. This is the first time that compounds designed to be mimics of ω-conotoxin GVIA and found to be active in the ^125^I-ω-conotoxin GVIA displacement assay have also been shown to block functional ion channels in a dose-dependent manner.

## 1. Introduction

Neuropathic pain, a pathology of the nervous system, is often highly debilitating and is thought to affect up to one-sixth of the world’s population [1]. There are numerous causes for the condition, including nerve damage resulting from surgery, trauma, infection and disease. This type of pain can be unresponsive to existing therapies. A combination of opioids, antidepressants and anticonvulsants is often prescribed, but usually only provides moderate pain relief and only in about 50% of cases [2,3], thus effective treatments for neuropathic pain represent a significant unmet medical need. Neuronal voltage-gated N-type calcium channels (Ca_v_2.2) are strongly implicated in chronic and neuropathic pain, but there are only three approved pain-blocking drugs that act on this channel; gabapentin, pregabalin and ziconotide [1]. Small molecule inhibitors of Ca_v_2.2 are potential leads for the treatment of neuropathic pain and have therefore been widely pursued in industry and academia [4,5,6]. Recent reports include those from Neuromed Pharmaceuticals (now Zalicus) [7,8,9,10], Abbott [11,12,13], Merck [14,15] and Janssen [16], with Neuromed’s NMED-160 reaching Phase II trials before being voluntarily withdrawn due to bioavailability issues. Reformulation of NMED-160, by Zalicus, gave Z160 which has now successfully completed Phase I trials. Phase II trials are due to commence in the second half of 2012 [10].

The ω-conotoxins are a family of calcium channel-blocking cystine knot peptides found in the venom of fish-hunting marine cone snails [17,18,19]. The most widely studied Ca_v_2.2 blockers from this class are ω-conotoxins GVIA, MVIIA and CVID. Ziconotide (also known as Prialt) is a synthetic version of ω-conotoxin MVIIA and is used in the clinic as a primary alternative to opioids for the management of intractable chronic pain. While this drug does not appear to lead to tolerance, its intrathecal delivery, narrow therapeutic index and side effect profile means that it suffers from high dropout rates and is far from an ideal treatment [20]. ω-Conotoxin CVID is more selective for Ca_v_2.2 channels and is expected to have greater therapeutic potential. This peptide reached Phase II in 2004 and is still being investigated under the name Leconotide [21,22]. Of the three peptides, ω-conotoxin GVIA has the highest affinity for the Ca_v_2.2 channel, but its virtual irreversible binding makes it unattractive as a therapeutic drug. The highly constrained nature of these peptides and the availability of SAR data make them ideal starting points for the development of peptidomimetics [23,24]. Mimics of all three of these peptides have been developed, beginning with the work of Horwell and co-workers at Parke-Davis, who prepared three-residue non-peptidic mimics of ω-conotoxin MVIIA based on a phloroglucinol core [25,26,27]. Subsequently, Lewis’ group developed a cyclic pentapeptide that mimics the action of ω-conotoxin CVID [28]. We have been investigating non-peptidic mimics of ω-conotoxin GVIA based on benzothiazole [29,30] and anthranilamide [31,32] cores. These mimics, which bear tyrosine, lysine and arginine side chain mimics projected from a central scaffold were designed based on Bartlett and Lauri’s Cα-Cβ bond vector philosophy, but—crucially—not involving automated scaffold retrieval but rather interactive de novo design [23,24,33].

**Figure 1:**
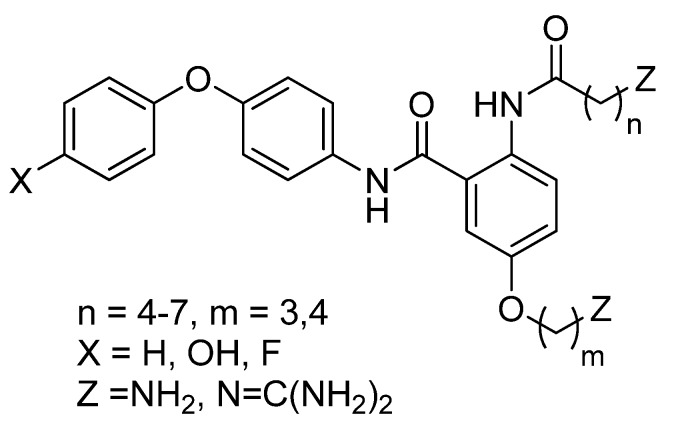
Previously described ω-conotoxin mimics based on an anthranilamide scaffold [32].

An investigation of the mimic based on the anthranilamide scaffold involved variation of the length of the side chains, the incorporation of two guanidino moieties, and two variations of the diphenyl ether substituent (see Figure 1) [32]. Key findings from this study were that; (a) typically diguanidino compounds [Z = N=(NH_2_)_2_] had binding activity in the 6–16 μM range in a radio-labelled ω-conotoxin GVIA displacement assay, whereas diamino compounds [Z = NH_2_] bound more weakly and; (b) strongest affinity was found with a fluorinated mimic [X = F]. 

**Figure 2:**
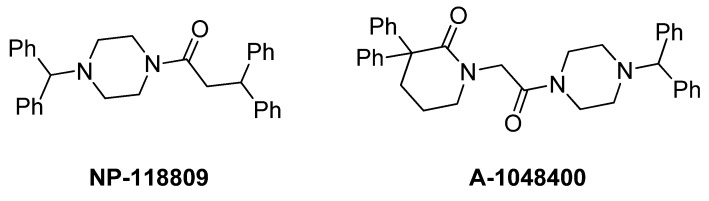
Recently published Ca_v_2.2 blockers bearing the diphenylpiperazine moiety.

**Figure 3:**
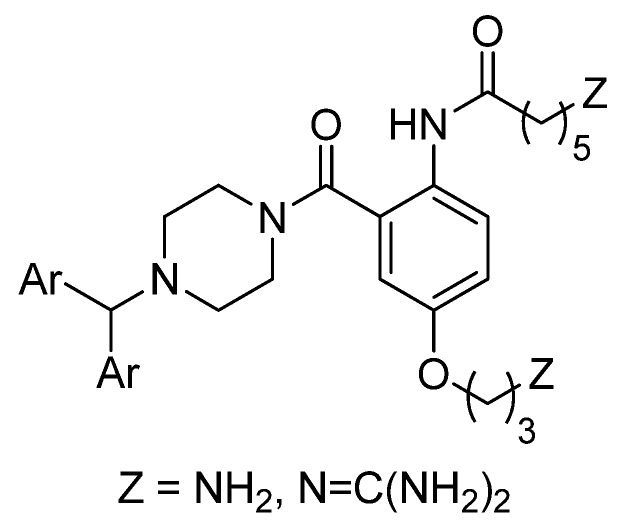
Analogues targeted in this study.

There are a number of compounds that effectively block voltage-gated calcium channels that bear the diphenylmethylpiperazine moiety [34]. Relevant to the current study are Neuromed’s NP-118809 (NMED-160) [7,8] and Abbott’s A-1048400 [11,12] shown in Figure 2. It was thus decided to prepare analogues of the anthranilamide-based conotoxin mimics where a diphenylmethylpiperazine moiety was incorporated in place of the phenoxyaniline substituent (Figure 3), and test their ability to block neuronal calcium channels in non-functional and, importantly, functional assays. 

## 2. Results and Discussion

### 2.1. Chemistry

**Scheme 1:**
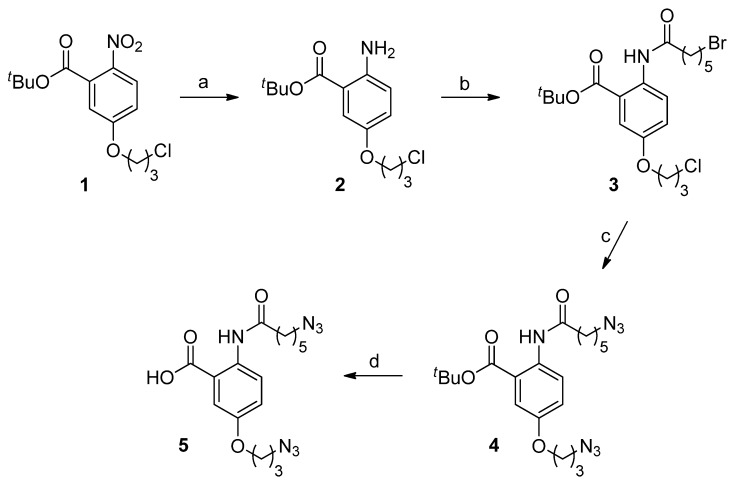
Reagents and conditions: (**a**) H_2_, Pd/C, ethanol; (**b**) 6-bromohexanoyl chloride, Triethylamine (Et_3_N), Dichloromethane (CH_2_Cl_2_); (**c**) Trimethylsilylazide (TMS-N_3_), Tetrabutylammonium fluoride (TBAF), Tetrahydrofuran (THF); (**d**) Trifluoroacetic acid (TFA), THF.

The previously described synthetic route to the anthranilamide-based mimics [32] was modified to allow incorporation of the diphenylmethylpiperazine moiety at a later stage in the synthesis, thus facilitating the preparation of a small library of compounds from a common, advanced precursor. This advanced precursor was the diazide (**5**), which was prepared in four steps from the previously reported nitro ester (**1**) [32], as shown in Scheme 1. This involved reduction of the nitro compound (**1**) to the aniline (**2**), acylation with 6-bromohexanoyl chloride to give the dihalide (**3**), conversion to the diazide (**4**) with *in situ*-formed tetra-*n*-butyl ammonium azide [35], and cleavage of the *t*-butyl ester.

**Scheme 2:**
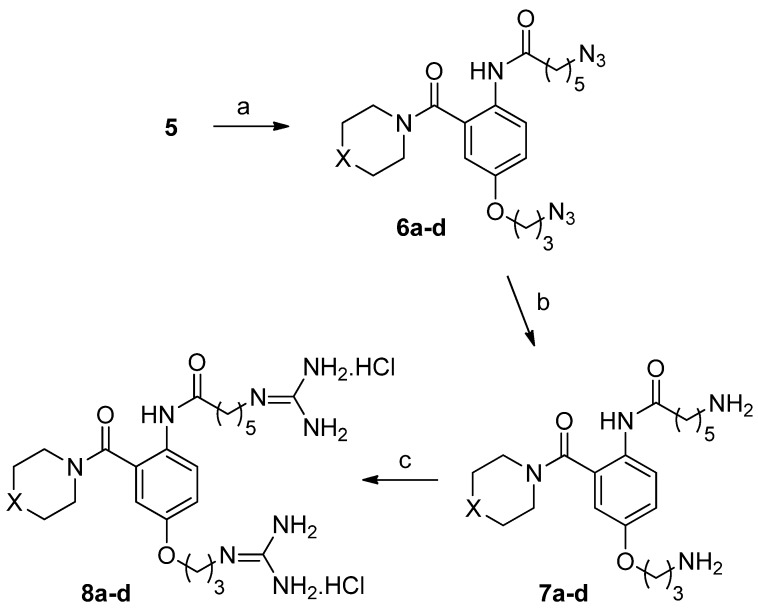
Reagents and conditions: (**a**) Dicyclohexylcarbodiimide (DCC), Hydroxybenzotriazole (HOBt), Dimethylformamide (DMF), cyclic secondary amine, Diisopropylethylamine (DIPEA), DMF/THF (1:1); (**b**) dithiothreitol, Et_3_N, methanol; (**c**) 1*H*-pyrazole-carboxamidine hydrochloride, DIPEA, DMF. For **6a**–**8a**, X = (Ph)_2_CH-N; for **6b**–**8b**, X = (4-F-Ph)_2_CH-N; for **6c**–**8c**, X = Ph-CH; for **6d**–**8d**, X = 1,3-Benzodioxolan-5-yl-CH_2_-N.

A small set of compounds was prepared in which the phenoxyaniline substituent of the original anthranilamide-based mimics was replaced with fluorinated and unfluorinated diphenylmethylpiperazine, 1,3-benzodioxomethylpiperazine and phenylmethylpiperidine substituents. The latter two substituents were chosen to test the importance of the presence of the diphenylmethyl functionality to Ca_v_2.2 activity. The required diamines (**7a**–**d**) and diguanidino compounds (**8a**–**d**) were prepared from diazido acid (**5**) in three steps, as shown in Scheme 2. This involved the use of a DCC-assisted amidation protocol described by Shpiro and Marquez [36] to give **6a**–**d**, reduction of the azide functionalities with dithiothreitol [37], to give **7a**–**d**, and guanidine formation by treatment with 1*H*-pyrazole-carboxamidine [38] to give **8a**–**d**.

### 2.2. Biology

#### 2.2.1. Radioligand Displacement Assay

The synthesized compounds, diamino (**7a**–**d**) and diguanidinium (**8a**–**d**), were initially evaluated for their ability to bind to rat brain Ca_v_2.2 channels using a previously described radioligand displacement assay [29,30,31,32,39], employing ^125^I-labelled ω-conotoxin GVIA. Although not a functional assay, the high selectivity of ω-conotoxin GVIA for Ca_v_2.2 channels means that compounds able to displace ^125^I-labelled ω-conotoxin GVIA from rat brain homogenate are likely to be functional inhibitors of Ca_v_2.2 channels. The results obtained with **7a**–**d** and **8a**–**d **are summarized in Table 1. 

**Figure 4:**
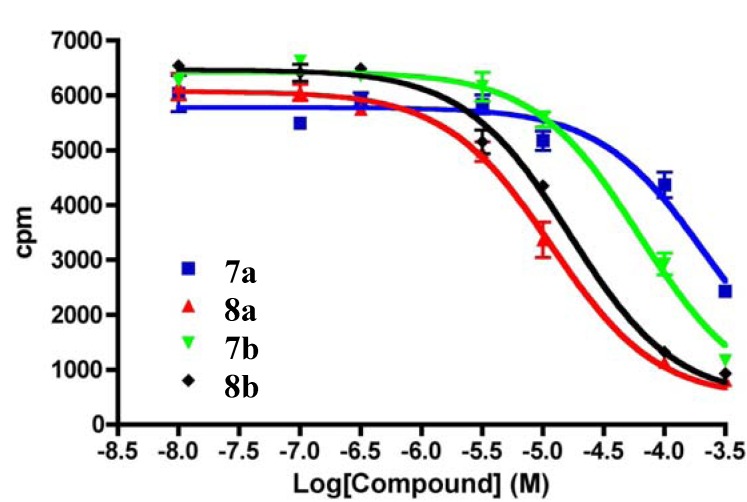
Dose-response curves for the displacement of ^125^I-GVIA from rat brain homogenate by diamino (**7a** and **7b**) and diguanidinium (**8a** and **8b**) compounds. cpm = counts per minute.

The affinities of the compounds that lack the diphenylmethyl functionality (**7c**, **7d**, **8c **and **8d**) were too weak to be measured by this method, whereas those that do possess that moiety (**7a**, **7b**, **8a **and **8b**) showed moderate to good binding (see Figure 4). As observed previously with this class of compound [32], the diguandino compounds (**8a **and **8b**) showed strongest binding, comparable to some of the best reported anthranilamides [32], determined by this method. Compounds **7a**, **7b**, **8a **and **8b **were therefore selected for follow-up functional assays.

**Table 1:** Ca_v_2.2 binding affinities of diamino (**7a**–**d**) and diguanidinium (**8a**–**d**) anthranilamides, as determined by displacement of ^125^I-GVIA from rat brain homogenate (95% confidence intervals are shown in parentheses).

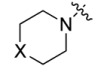	**Diamino compound**	**EC_50_ Ca_v_2.2 (μM)**	**Diguanidinium compound**	**EC_50_ Ca_v_2.2 (μM)**
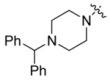	**7a**	214 (160–290)	**8a**	12 (9–15)
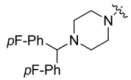	**7b**	65 (50–83)	**8b**	16 (13–20)
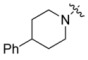	**7c**	weak	**8c**	weak
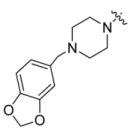	**7d**	weak	**8d**	weak

#### 2.2.2. Inhibition of Neuroblastoma Intracellular Calcium Responses, Determined by Fluorescence Measurement of Calcium Flux

**Figure 5:**
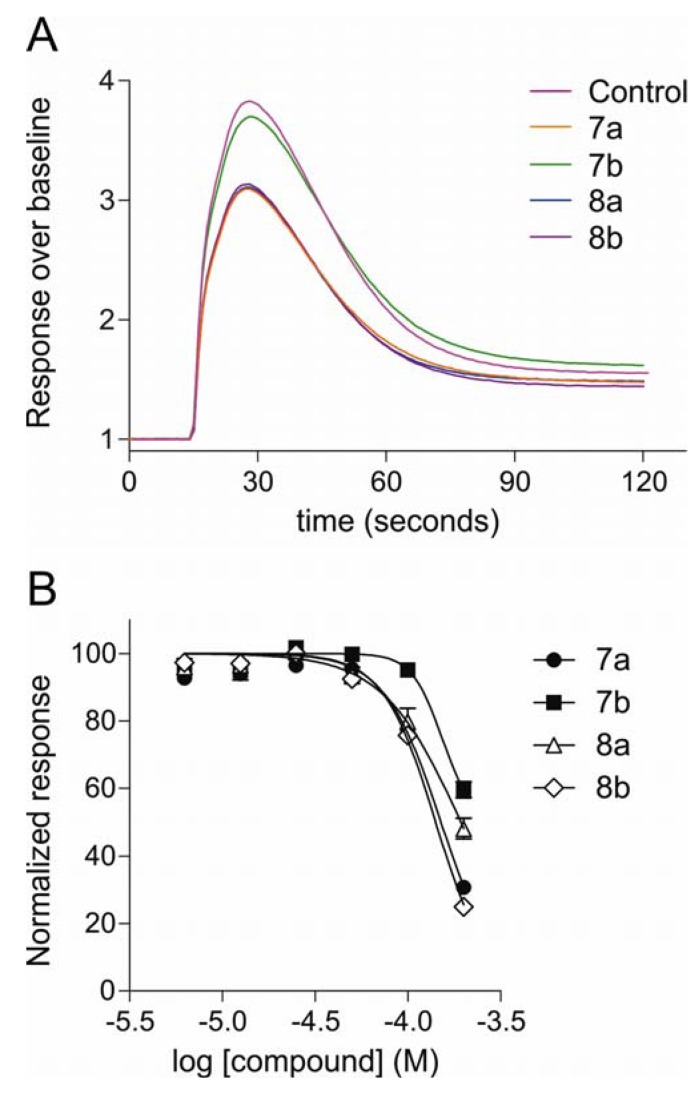
Inhibition of calcium responses from SH-SY5Y human neuroblastoma cells by diamino (**7a** and **7b**) and diguanidinium (**8a** and **8b**) compounds. (**A**) Calcium channel-mediated Ca^2+^ transients were partially inhibited by pre-treatment with compounds **7a**, **8a** and **8b** at 100 µM. (**B**) Dose-response curves for functional inhibition of calcium responses.

The abilities of the diamino (**7a** and **7b**) and diguanidinium (**8a** and **8b**) compounds to inhibit intracellular calcium responses in SH-SY5Y human neuroblastoma cells, in the presence of the L-type calcium channel blocker nifedipine were assessed using a high-throughput Ca^2+^ fluorescence assay. It was found that Ca^2+^ ion channel responses elicited by KCl-mediated depolarization were partially inhibited by the test compounds at concentrations which did not produce fluorescence addition artefacts (100 µM; Figure 5A), with compound **7b** being less efficacious (4.9 ± 1.8% inhibition) than compounds **7a** (23.1 ± 1.5% inhibition), **8a** (20.3 ± 4.0% inhibition) and **8b** (24.4 ± 1.5% inhibition). While these inhibitory effects are not strong, dose-dependent inhibition was observed (Figure 5B) which allowed the estimation of IC50’s for the functional inhibition of intracellular calcium responses in SH-SY5Y human neuroblastoma cells. These results are shown in Table 2. The estimated IC50s from these functional, whole-cell experiments are up to an order of magnitude weaker than those obtained from the radioligand displacement assay. A similar shift in potency is observed with the ω-conotoxins, whose effectiveness is reduced in the presence of physiological Ca^2+^ levels [40] and auxiliary subunits [41], which are likely to dissociate from the Ca_v_ α-subunit in membrane preparations used in radioligand displacement assays. It is also plausible that the decreased potency of the test compounds in the functional assays, in addition to effects of co-expressed auxiliary subunits, may have been influenced by relatively short incubation times in the presence of extracellular divalent cations, which has been reported to affect the on-rate of ω-conotoxin block.

**Table 2:** Estimated IC_50_ values (μM; mean ± SEM) for the functional inhibition of calcium channels by compounds **7a**, **7b**, **8a** and **8b**.

Compound	SH-SY5Y	HEK293
	neuroblastoma cells	*hCa_v_2*. *2 + β3 + α2δ1*
**7a**	160 ± 3.6	232 ± 23
**7b**	286 ± 66	288 ± 49
**8a**	206 ± 50	299 ± 43
**8b**	156 ± 10	156 ± 21

Despite giving comparable radioligand displacement assay results to the best previously reported anthranilamides (see Figure 1) [32], the new diphenylmethylpiperazine-substituted compounds (**7a**, **8a** and **8b**) showed superior inhibition of intracellular calcium responses in SH-SY5Y cells (data not shown) and hence can be considered an improvement on previous designs. 

#### 2.2.3. Patch-Clamp Electrophysiology Experiments with HEK293 Cells Expressing Ca_v_2.2 Calcium Channels

**Figure 6:**
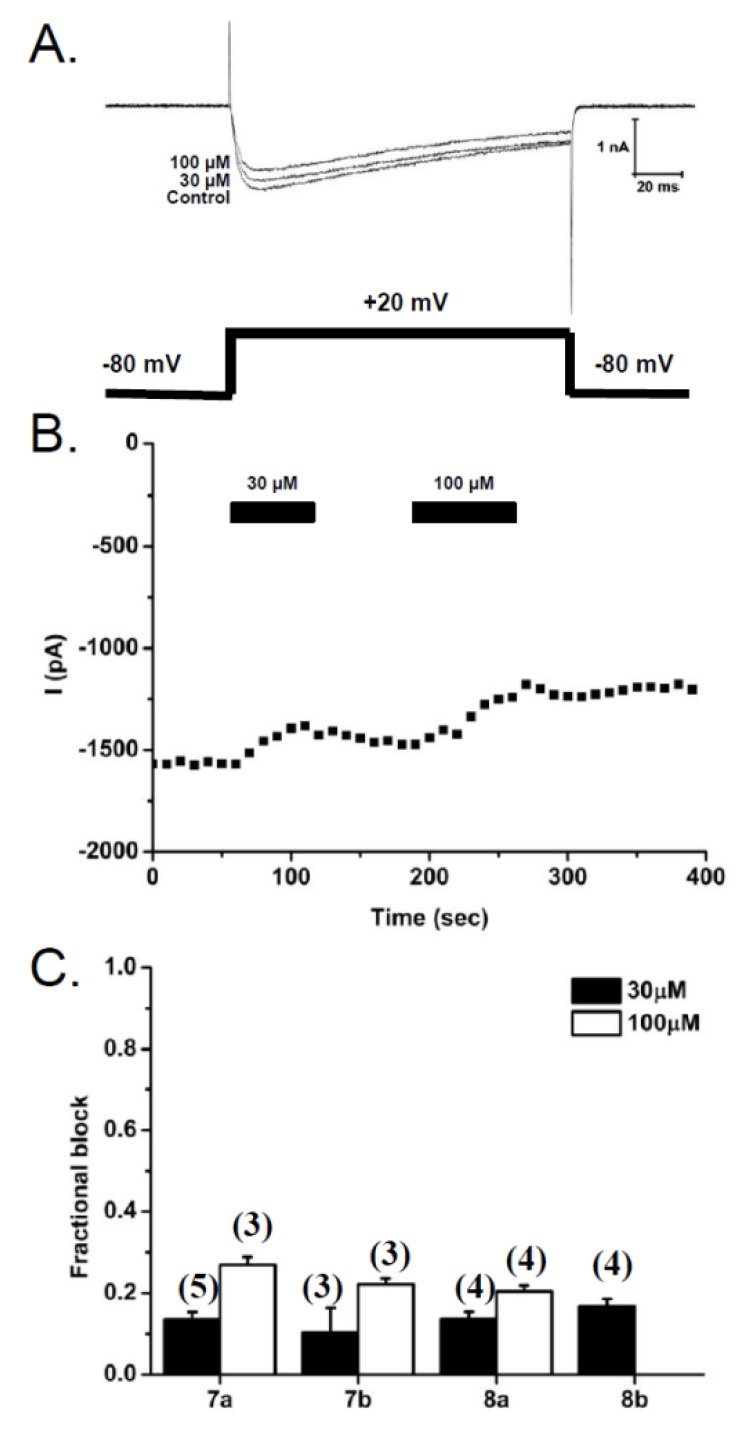
Effects of diamino (**7a** and **7b**) and diguanidinium (**8a** and **8b**) anthranilamides on Ca_v_2.2 currents in HEK293 cells. (**A**) Superimposed whole-cell calcium currents obtained from HEK293 cells stably expressing N-type calcium channels (*hCa_v_2.2* + *β3* + *α2δ1*), with depolarizing voltage steps from a holding potential of −80 mV to a test potential of +20 mV (150 ms). Representative currents are shown prior to compound **7a** application (Control) and in the presence of compound **7a** at two concentrations (30 and 100 µM). (**B**) Time course of inhibition of peak inward current in response to 30 and 100 µM compound **7a**. (**C**) Bar graph of the relative inhibition of peak current amplitude (1 −I_compound_/I_control_) for each compound at concentrations of 30 and 100 µM (number of experiments in parentheses).

SH-SY5Y human neuroblastoma cells contain a number of calcium channel subtypes [42,43,44,45]. To specifically measure the effect of the diamino (**7a** and **7b**) and diguanidinium (**8a** and **8b**) anthranilamides on N-type (Ca_v_2.2) calcium channel currents, electrophysiological patch-clamp studies were carried out on HEK293 cells stably expressing human Ca_v_2.2 (*a1 *+ *β3 *+ *α2δ1* subunits). Compounds **7a**, **7b** and **8a** were each tested at two concentrations (30 and 100 µM), whereas **8b** was only tested at 30 µM. Representative traces obtained prior to compound application (Control) and following application of compound **7a** at 30 and 100 µM are shown in Figure 6A. The time course of washing in and out of compound **7a** is shown in Figure 6B, with peak current amplitude being measured at 10 s intervals. All four compounds showed a modest inhibition of the calcium current amplitude; 10%–17% at 30 µM and 20%–27% at 100 µM (Figure 6C), while exhibiting no shift in the G–V relationship or changes to channel inactivation. To determine an approximate IC_50_ value, the data were fit assuming that these compounds completely inhibited the calcium currents, with a Hill coefficient of −1. This gave the predicted half-maximal inhibition (IC_50_’s) shown in Table 2 and are consistent with those obtained using the FLIPR-Ca^2+^ assay in SH-SY5Y cells. The estimated IC_50_ values for **8b** obtained from the two techniques are in close agreement and, of all the compounds examined in this study, this compound appears to most effectively block Ca_v_2.2 calcium channels.

## 3. Experimental Section

### 3.1. Chemistry

#### 3.1.1. General Experimental Procedures

Starting materials and reagents were purchased from Sigma-Aldrich and used without purification. Tetrahydrofuran (THF) was distilled under a nitrogen atmosphere from sodium benzophenone ketal. Dry DCM was obtained after drying over 3 Å sieves. Methanol and DMF were obtained by passage through two alumina columns on the Solvent Dispensing System built by J. C. Meyer and based on the original design by Grubbs and co-workers [46]. Solutions were dried over anhydrous magnesium sulfate (MgSO_4_) or sodium sulfate (Na_2_SO_4_). Normal phase flash chromatography was performed on Merck silica gel No. 9385 and reverse phase chromatography was performed using a C18 Chromatorex^®^ DM1020T column (30 × 40 mm). Spectra were recorded on a Bruker Av200 or Av400 spectrometer. Unless otherwise stated, proton (^1^H) NMR spectra were acquired at 200 MHz and carbon (^13^C) NMR spectra at 50 MHz. NMR spectra were referenced to residual solvent peak [chloroform (δ_H_ 7.26, δ_C_ 77.0), methanol (δ_H_ 4.87, 3.30, δ_C_ 49.86)]. The units for all coupling constants (*J*) are in hertz (Hz). Low resolution mass spectra were recorded on a Micromass Platform spectrometer or VG Platform spectrometer. Accurate mass determinations were carried out at high resolution on an Agilent G1969A LC-TOF system, with reference and mass correction at 4000 V capillary voltage for ESI. 

#### 3.1.2. Synthesis

##### *tert*-Butyl-2-amino-5-(3-chloropropoxy)benzoate (2)

To a vigorously stirred flask containing *tert*-butyl-5-(3-chloropropoxy)-2-nitrobenzoate [32] **1** (5.17 g, 16.4 mmol) in ethanol (100 mL), was added 10% wt palladium on carbon (0.17 g). The reaction mixture was flushed with hydrogen (3×) and then stirred under an atmosphere of hydrogen for 15 h. The reaction mixture was filtered through Celite, the Celite thoroughly rinsed with methanol and the combined organics concentrated *in vacuo*. The residue was taken up in diethyl ether (150 mL) and washed with water and brine, then dried (MgSO_4_) and concentrated to afford the title compound as an oil (4.18 g, 89%). This compound was used in subsequent transformations without further purification. δ_H_ (CDCl_3_) 1.62 (s, 9H), 2.25 (m, 2H), 3.75 (t, *J *= 6.2 Hz, 2H), 4.15 (t, *J *= 6.0 Hz, 2H), 7.17 (dd, *J *= 3.0, 8.8 Hz, 1H), 7.52 (d, *J *= 3.0 Hz, 1H), 7.90 (d, *J *= 8.8 Hz, 1H); δ_C_ (CDCl_3_) 28.1, 31.9, 41.1, 64.9, 84.5, 117.3, 119.9, 123.8, 125.7, 127.0, 158.0, 166.0; *m/z* (EI^+^): *m/z* (%): 285.1 (30) [M]^+^, 229.0 (100) [M − C_4_H_9_]^+^.

##### *tert*-Butyl-2-(6-bromohexanamido)-5-(3-chloropropoxy)benzoate (3)

6-Bromohexanoyl chloride (2.1 mL, 14 mmol) was added dropwise to the aniline **2** (3.72 mg, 13.0 mmol) and triethylamine (2.7 mL, 19 mmol) dissolved in dry DCM (70 mL). The reaction mixture was stirred at RT for 20 h and then poured into 2 M HCl (50 mL). The layers were separated and the aqueous phase was extracted with DCM. The combined organic phases were washed with saturated sodium bicarbonate and brine, then dried (MgSO_4_) and concentrated *in vacuo *to afford the amide **3** as an oil (6.02 g, quant.); δ_H_ (CDCl_3_) 1.49–1.99 (complex, 6H), 1.61 (s, 9H), 2.23 (m, 2H), 2.44 (t, *J *= 7.2 Hz, 2H), 3.42 (t, *J *= 6.8 Hz, 2H), 3.75 (t, *J *= 6.2 Hz, 2H), 4.11 (t, *J *= 6.0 Hz, 2H), 7.08 (dd, *J *= 3.0, 9.2 Hz, 1H), 7.47 (d, *J *= 3.0 Hz, 1H), 8.62 (d, *J *= 9.2 Hz, 1H), 10.93 (br s, 1H). δ_C_ (CDCl_3_) 24.6, 27.7, 28.2, 32.2, 32.5, 33.5, 38.2, 41.4, 64.7, 82.6, 116.3, 117.7, 120.2, 121.8, 135.4, 153.3, 167.3, 171.3; *m/z* (ESI^+^, 50 eV): *m/z* (%): 486.0 (100) [M + Na]^+^, 484.0 (70), 464 [M + H]^+^, 462.0 (20).

##### *tert*-Butyl-2-(6-azidohexanamido)-5-(3-azidopropoxy)benzoate (4)

The diazide **4** was prepared from the dihalide **3** (5.92 g, 12.8 mmol) by treatment with trimethylsilylazide (95% purity, 5.4 mL, 39 mmol) and tetra-*n*-butylammonium fluoride (1.0 M in THF, 38.4 mL, 38.4 mmol) in THF following the method of Takaya [35]. Care was taken to ensure the diazide was not subjected to shock or heat. The diazide **4** was obtained as a light brown oil which was purified by flash chromatography (20: 1 DCM/MeOH) to afford a cream-colored solid (4.09 g, 74%). δ_H_ (CDCl_3_) 1.40–1.85 (complex, 6H), 1.59 (s, 9H), 2.02 (m, 2H), 2.42 (t, *J *= 7.2 Hz, 2H), 3.26 (t, *J *= 6.6 Hz, 2H), 3.50 (t, *J *= 6.6 Hz, 2H), 4.02 (t, *J *= 5.8 Hz, 2H), 7.05 (dd, *J *= 3.2, 9.2 Hz, 1H), 7.44 (d, *J *= 3.2 Hz, 1H), 8.60 (d, *J *= 9.2 Hz, 1H), 10.92 (br s, 1H); δ_C_ (CDCl_3_) 25.0, 26.3, 28.1, 28.6, 28.7, 38.2, 48.1, 51.2, 64.9, 82.6, 116.2, 117.6, 120.2, 121.8, 135.4, 153.2, 167.2, 171.3; *m/z* (ESI^+^, 50 eV): *m/z* (%): 454.6 (15) [M + Na]^+^, 432.7 (100) [M + H]^+^. 

##### 2-(6-Azidohexanamido)-5-(3-azidopropoxy)benzoic acid (5)

To a solution of **4 **(1.31 g, 3.05 mmol) in dry DCM (10 mL) was added TFA (10 mL) dropwise. After stirring the reaction mixture at RT for 4 h it was poured into water (20 mL) and extracted with DCM (2 × 10 mL). The organic phase was dried, and concentrated *in vacuo* to yield the title compound as a solid (1.14 g, 99%). δ_H_ (CDCl_3_) 1.43–1.87 (complex, 6H), 2.05 (m, 2H), 2.48 (t, *J *= 7.2 Hz, 2H), 3.28 (t, *J *= 6.6 Hz, 2H), 3.52 (t, *J *= 6.4 Hz, 2H), 4.06 (t, *J *= 5.8 Hz, 2H), 7.16 (dd, *J *= 3.0, 9.2 Hz, 1H), 7.61 (d, *J *= 3.0 Hz, 1H), 8.64 (d, *J *= 9.2 Hz, 1H), 10.86 (br s, 1H); δ_C_ (CDCl_3_) 25.1, 26.2, 28.5, 28.7, 38.1, 48.1, 51.2, 65.0, 116.0, 116.6, 121.6, 122.1, 172.2, 135.3, 153.7, 170.9; *m/z* (ESI^+^, 50 eV): *m/z* (%): 398.6 (100) [M + Na]^+^, 376.5 (70) [M + H]^+^.

##### 6-Azido-*N*-(4-(3-azidopropoxy)-2-(4-benzhydrylpiperazine-1-carbonyl)phenyl)hexanamide (6a)

The title compound was prepared following an amidation procedure used by Marquez and Shpiro [36]. To a solution of the diazide **5 **(120 mg, 0.33 mmol) in anhydrous THF and DMF (1:1, 3.2 mL) was added DCC (100 mg, 0.49 mmol). After the reaction mixture was stirred at RT for 15 min, HOBt (70 mg, 0.49 mmol) and 4-DMAP (8 mg, 0.06 mmol) were added. After 1.5 h, 1-benzhydrylpiperazine (170 mg, 0.65 mmol) and DIPEA (0.17 mL, 0.98 mmol) were sequentially added. After stirring at RT for 24 h, the mixture was filtered, the precipitate washed with DCM and solvent was removed *in vacuo*. Purification by flash chromatography (3:1 PET spirit 40–60/ethyl acetate) gave the title compound **6a** as a solid (130 mg, 66%); δ_H_ (CDCl_3_) 1.40–1.80 (complex, 6H), 2.01 (m, 2H), 2.32 (t, *J *= 7.2 Hz, 2H), 2.40 (br s, 4H), 3.30 (t, *J *= 6.6 Hz, 2H), 3.48 (t, *J *= 6.6 Hz, 2H), 3.71 (br s, 4H), 3.98 (t, *J *= 6.0 Hz, 2H), 4.26 (s, 1H), 6.70 (d, *J *= 2.8 Hz, 1H), 6.89 (dd, *J *= 3.0, 9.0 Hz, 1H), 7.15–7.43 (m, 10H), 8.01 (d, *J *= 8.0 Hz, 1H), 8.49 (br s, 1H); δ_C_ (CDCl_3_) 25.1, 26.3, 28.6, 28.7, 37.3, 48.1, 51.2, 51.9 (br), 52.1 (br), 65.0, 75.9, 113.4, 116.0, 125.0, 126.5, 127.2, 127.8, 128.6, 129.6, 141.9, 154.4, 168.3, 171.1; *m/z* (ESI^+^, 50 eV): *m/z* (%): 632.3 (10) [M + Na]^+^, 610.3 (50) [M + H]^+^. 

##### 6-Azido-*N*-(4-(3-azidopropoxy)-2-(4-(bis(4-fluorophenyl)methyl)piperazine-1-carbonyl)phenyl)hexanamide (6b)

The title compound **6b** was prepared from **5 **and 1-(bis(4-fluorophenyl)methyl)piperazine according to the procedure used to prepare **6a**, except that the reaction mixture was stirred at RT for 56 h. Purification by flash chromatography (2:1 PET spirit 40–60/ethyl acetate) gave **6b** (140 mg, 85%). δ_H_ (CDCl_3_) 1.39–1.79 (m, 6H), 2.01 (m, 2H), 2.31 (t, *J *= 7.6 Hz, 2H), 2.36 (br s, 4H), 3.29 (t, *J *= 6.8 Hz, 2H), 3.48 (t, *J *= 6.4 Hz, 2H), 3.73 (br s, 4H), 3.98 (t, *J *= 6.0 Hz, 2H), 4.24 (s, 1H), 6.68 (d, *J *= 2.8 Hz, 1H), 6.89 (dd, *J *= 2.8, 9.0 Hz, 1H), 6.92–7.02 (m, 4H), 7.29–7.36 (m, 4H,), 7.95 (d, *J *= 9.0 Hz, 1H), 8.50 (br s, 1H); δ_C_ (CDCl_3_) 25.1, 26.3, 28.6, 28.7, 37.2, 48.1, 51.2, 51.7 (br), 65.0, 74.2, 113.4, 115.6 (d, *J* = 21.6 Hz), 116.0, 125.3, 126.8, 129.1, 129.2 (d, *J* = 7.9 Hz), 137.4 (d, *J* = 3.0 Hz), 154.5, 161.9 (d, *J* = 246.5 Hz), 168.4, 171.2; *m/z* (ESI^+^, 50 eV): *m/z* (%): 668.3 (10) [M + Na]^+^, 646.3 (35) [M + H]^+^.

##### 6-Azido-*N*-(4-(3-azidopropoxy)-2-(4-phenylpiperidine-1-carbonyl)phenyl)hexanamide (6c)

The title compound **6c** was prepared from **5** and 4-phenylpiperidine according to the procedure used to prepare **6a**. Purification by flash chromatography (1:1 PET spirit 40–60/ethyl acetate) gave **6c** (190 mg, 93%); δ_H_ (CDCl_3_) 1.37–2.00 (methylene envelope, 10H), 2.02 (m, 2H), 2.32 (t, *J *= 6.0 Hz, 2H), 2.78 (m, 1H), 3.00 (br s, 2H), 3.25 (t, *J *= 6.0 Hz, 2H), 3.49 (t, *J *= 6.0 Hz, 2H), 4.01 (t, *J *= 6.0 Hz, 2H), 4.80 (br s, 2H), 6.76 (d, *J *= 2.0 Hz, 1H), 6.90 (dd, *J *= 2.0, 6.0 Hz, 1H), 7.17–7.32 (m, 5H), 7.90 (d, *J *= 8.0 Hz, 1H), 8.63 (s, 1H); δ_C_ (CDCl_3_) 24.8, 25.0, 26.2, 28.5, 28.6, 33.3 (br), 33.8, 37.0, 42.3, 48.0, 51.1, 64.9, 113.0, 115.8, 125.4, 126.5, 127.6, 128.5, 129.1, 144.6, 154.6, 168.4, 171.1; *m/z* (ESI^+^, 50 eV): *m/z* (%): 541.3 (100) [M + Na]^+^, 519.3 (20) [M + H]^+^.

##### 6-Azido-*N*-(4-(3-azidopropoxy)-2-(4-((3a,7a-dihydrobenzo[*d*][1,3]dioxol-5-yl)methyl)piperazine-1-carbonyl)phenyl)hexanamide (6d)

The title compound **6d** was prepared from **5 **and 1-piperonylpiperazine according to the procedure used to prepare **5**. Purification by flash chromatography (5:1 PET spirit 40–60/ethyl acetate) gave **6d** (200 mg, 54%); δ_H_ (400 MHz, CDCl_3_) 1.43 (m, 2H), 1.59–1.74 (methylene envelope, 4H), 2.02 (m, 2H), 2.30 (t, *J*= 7.6 Hz, 2H), 2.41 (br s, 2H), 2.47 (br s, 2H), 3.27 (t, *J *= 6.8 Hz, 2H), 3.44 (s, 2H), 3.49 (t, *J *= 6.8Hz, 2H), 3.75 (br s, 4H), 4.00 (t, *J *= 6.0 Hz, 2H), 5.92 (s, 2H), 6.70–6.72 (m, 3H), 6.83 (s, 1H), 6.89 (dd, *J *= 2.8, 8.8 Hz, 1H), 7.91 (d, *J *= 8.8 Hz, 1H), 8.52 (s, 1H); δ_C_ (100 MHz, CDCl_3_) 25.1, 26.3, 28.6, 28.7, 37.2, 48.1, 51.2, 52.6 (br), 53.0 (br), 62.4, 65.0, 101.0, 108.0, 109.4, 113.3, 116.1, 122.3, 125.4, 127.0, 129.4, 130.9, 146.9, 147.8, 154.6, 168.4, 171.2; HRMS (ES^+^) [M + H]^+^ calcd for C_28_H_36_N_9_O_5_ 578.2839 found 578.2829.

##### 6-Amino-*N*-(4-(3-aminopropoxy)-2-(4-benzhydrylpiperazine-1-carbonyl)phenyl)hexanamide (7a)

A solution of **6a** (140 mg, 0.22 mmol) in methanol (4.5 mL) was treated sequentially with dithiothreitol (20 mg, 1.3 mmol) and TEA (0.1 mL, 0.9 mmol). After the reaction mixture was stirred at room temperature for 20 h, the solvent was removed *in vacuo*. Purification by reverse phase chromatography [elution with methanol/water (90:10, 60 mL), followed by methanol (75 mL) and then acetonitrile (30 mL)] gave **7a** (60 mg, 52%); δ_H_ (methanol-*d*_4_) 1.35–1.60 (complex, 4H), 1.69 (m, 2H), 1.91 (m, 2H), 2.33 (complex, 4H), 2.45 (m, 2H), 2.67 (br t, *J *= 6.8 Hz, 2H), 2.82 (br t, *J *= 6.9 Hz, 2H), 3.38 (br s, 2H), 3.71 (br s, 2H), 4.04 (br t, *J *= 6.2 Hz, 2H), 4.30 (s, 1H), 6.82 (d, *J *= 2.8 Hz, 1H), 6.98 (dd, *J *= 2.8, 8.8 Hz, 1H), 7.18–7.29 (m, 7H,), 7.43 (d, *J *= 6.9 Hz, 4H); δ_C_ (methanol-*d*_4_) 26.8, 27.6, 33.2, 33.3, 37.2, 39.6, 42.3, 43.2, 49.9, 52.6, 53.1, 67.5, 77.2, 114.1, 117.2, 128.3, 129.0, 129.1, 129.7, 133.9, 143.7, 158.3, 170.0, 171.2; *m/z* (ESI^+^, 50 eV): *m/z* (%): 558.3 (100) [M + H]^+^, 570.3 (30) [M + Na]^+^. HRMS (ESI^+^) [M + H]^+^ calcd for C_33_H_44_N_5_O_3_ 558.3444 found 558.3460. 

##### 6-Amino-*N*-(4-(3-aminopropoxy)-2-(4-(bis(4-fluorophenyl)methyl)piperazine-1-carbonyl)phenyl)hexanamide (7b)

The diamine **7b** was prepared from **6b** according to the procedure used to prepare **7a**. The product was isolated as an oil (90 mg, 75%). δ_H_ (methanol-*d*_4_) 1.36–1.75 (methylene envelope, 6H), 1.91 (m, 2H), 2.31 (t, *J *= 7.4 Hz, 2H), 2.42 (br s, 4H), 2.66 (t, *J *= 7.0 Hz, 2H), 2.80 (t, *J *= 7.0 Hz, 2H), 3.38 (br s, 2H), 3.70 (br s, 2H), 4.03 (t, *J *= 6.0 Hz, 2H), 4.35 (s, 1H), 6.81 (d, *J *= 2.6 Hz, 1H), 6.94–7.05 (m, 5H), 7.26 (d, *J *= 8.8 Hz, 1H), 7.38–7.45 (m, 4H); δ_C_ (methanol-*d*_4_) 26.7, 27.5, 32.9, 33.0, 37.1, 39.5, 42.1, 43.1, 52.4, 52.7, 67.5, 75.2, 114.1, 116.3 (d, *J *= 21.5 Hz), 117.0, 129.0, 130.7 (d, *J* = 7.9 Hz), 133.7, 139.4 (d, *J* = 3.1 Hz), 158.2, 163.3 (d, *J* = 244.3 Hz), 169.9, 175.0; *m/z* (ESI^+^, 50 eV): *m/z* (%): 594.3 (100) [M + H]^+^. HRMS (ESI^+^) [M + H]^+^ calcd for C_33_H_42_N_5_O_3_F_2_ (M + H)^+^ 594.3256 found 594.3259.

##### 6-Amino-*N*-(4-(3-aminopropoxy)-2-(4-phenylpiperidine-1-carbonyl)phenyl)hexanamide (7c)

The diamine **7c** was prepared from **6c **according to the procedure used to prepare **7a**. Purification by reverse phase chromatography [elution with methanol/water (80:20)] gave **7c** as an oil (120 mg, 99%); δ_H_ (400 MHz, methanol-*d*_4_) 1.39–1.60 (methylene envelope, 4H), 1.72 (m, 4H), 1.95 (m, 2H), 2.37 (m, 2H), 2.68 (m, 2H), 2.80–2.91 (complex, 5H), 3.17 (br s, 2), 3.76 (br d, *J *= 13.2 Hz, 1H), 4.07 (t, *J *= 6.0 Hz, 2H), 4.74 (d, *J *= 12.0 Hz, 1H), 6.90 (s, 1H), 7.01 (dd, *J *= 2.4, 8.4 Hz, 1H),7.18–7.43 (m, 6H); δ_C_ (methanol-*d*_4_) 25.3, 26.1, 31.1, 31.4, 32.7, 33.3, 35.7, 38.1, 40.6, 42.2, 66.1, 112.5, 115.5, 126.1, 126.4, 126.8, 127.7, 128.2, 132.8, 145.3, 156.9, 168.5, 173.6; *m/z* (ESI^+^, 50 eV): *m/z* (%): 467.3 (100) [M + H]^+^, 489.3 (55) [M + Na]^+^. 

##### 6-Amino-*N*-(4-(3-aminopropoxy)-2-(4-(benzo[*d*][1,3]dioxol-5-ylmethyl)piperazine-1-carbonyl)phenyl)hexanamide (7d)

The diamine **7d** was prepared from **6d **according to the procedure used to prepare **7a**. The product was isolated as an oil (90 mg, 61%); δ_H_ (methanol-*d*_4_) 1.37–1.76 (methylene envelope, 6H), 1.94 (m, 2H), 2.32 (t, *J *= 7.2 Hz, 2H), 2.39 (br s, 2H), 2.50 (br s, 2H), 2.70 (t, *J *= 6.8 Hz, 2H), 2.84 (t, *J *= 6.8 Hz, 2H), 3.36 (br s, 2H), 3.47 (s, 2H), 3.70 (br s, 2H), 4.06 (t, *J *= 6.0 Hz, 2H), 5.91 (s, 2H), 6.75 (br s, 2H), 6.82 (br s, 2H), 7.00 (dd, *J *= 2.8, 8.8 Hz, 1H), 7.28 (d, *J *= 8.8 Hz, 1H); δ_C_ (methanol-*d*_4_) 25.3, 26.1, 31.2, 31.5, 35.7, 38.1, 40.6, 41.4, 52.0, 52.3, 62.0, 66.1, 100.9, 107.4, 109.2, 112.6, 115.7, 122.4, 126.8, 127.7, 130.8, 132.4, 147.0, 147.8, 156.9, 168.5, 173.7; *m/z* (ESI^+^, 50 eV): *m/z* (%): 526.4 (20) [M + Na]^+^, 477.3 (30) [M + H]^+^. HRMS (ES^+^) [M + H]^+^ calcd for C_28_H_39_N_5_O_5_ 526.3029 found 526.3018.

##### *N*-2(4-Benzhydrylpiperazine-1-carbonyl)-4-(3-guanidinopropoxy)phenyl-6-guanidinohexanamide dihydrochloride (8a)

The diguanidinium compound **8a** was prepared following the general procedure of Bernatowitcz *et al. *[21]. To the amine **7a **(50 mg, 0.09 mmol) in DMF (0.2 mL) was added 1*H*-pyrazole-carboxamidine hydrochloride (30 mg, 0.17 mmol) and DIPEA (0.03 mL, 0.17 mmol) and the reaction mixture was stirred at RT for 68 h. The solvent was then removed *in vacuo* and the residue triturated with diethyl ether to give a solid that was recrystallized from ether/methanol to afford **8a **as a glassy semisolid (45 mg, 74%); δ_H_ (methanol-*d*_4_) 1.49 (m, 2H), 1.67 (m, 4H), 2.04 (m, 2H), 2.32–2.46 (complex, 6H), 3.18-3.42 (methylene envelope, 6H), 3.71 (br s, 2H), 4.07 (t, *J *= 5.6 Hz, 2H), 4.32 (s, 1H), 6.86 (d, *J *= 2.6 Hz, 1H), 7.02 (dd, *J *= 2.8, 8.8 Hz, 1H), 7.11–7.33 (m, 8H), 7.43 (br d, *J *= 7.0 Hz, 3H); δ_C_ (methanol-*d*_4_) 26.4, 27.2, 29.6, 36.9, 39.6, 42.4, 43.2, 52.6, 53.0, 66.6, 77.2, 114.3, 117.2, 121.4, 128.3, 128.5, 129.0, 129.7, 133.9, 143.7, 158.1, 158.7, 158.8, 170.0, 175.0; *m/z* (ESI^+^, 50 eV): *m/z* (%): 642.5 (100) [M + H]^+^. HRMS (ESI^+^) [M + H]^+^ calcd for C_35_H_48_N_9_O_3_ 642.3880 found 642.3885.

##### *N*-2-(4-(Bis(4-fluorophenyl)methyl)piperazine-1-carbonyl)-(4-(3-guanidinopropoxy)phenyl)guanidinohexanamide dihydrochloride (8b)

The diguanidinium compound **8b** was prepared from the diamine **7b** according to the method used to prepare **8a**. The product **8b** was obtained as a semiglassy solid (90 mg, 84%); δ_H_ (methanol-*d*_4_) 1.40–1.55 (methylene envelope, 2H) 1.55–1.80 (methylene envelope, 4H), 2.04 (t, *J *= 6.0 Hz, 2H), 2.25–2.45 (methylene envelope, 6H), 3.20 (t, *J *= 6.0 Hz, 2H), 3.30–3.42 (methylene envelope, 6H), 3.70 (br s, 2H), 4.06 (t, *J *= 6.0 Hz, 2H), 4.38 (br s, 1H), 6.86 (d, *J *= 4.0 Hz, 1H), 7.01 (m, 5H), 7.26 (d, *J *= 10.0 Hz, 1H), 7.39–7.46 (m, 5H); δ_C_ (methanol-*d*_4_) 25.0, 25.8, 28.2, 35.5, 38.1, 40.9, 41.8, 51.0, 51.3, 65.2, 73.7, 113.0, 114.9 (d, *J* = 21.6 Hz), 115.8, 127.0, 127.6, 129.3 (d, *J* = 8.0 Hz), 132.4, 137.9 (d, *J* = 3.0 Hz), 156.6, 157.2, 157.4, 161.9 (d, *J* = 244.5 Hz), 168.5, 173.6; *m/z* (ESI^+^, 50 eV): *m/z* (%): 714.4 (10) [M + HCl]^+^, 678.4 (50) [M + H]^+^. HRMS (ESI^+^) [M + 2H + Cl]^+^ calcd for C_35_H_47_N_9_O_3_F_2_Cl 714.3458 found 714.3462; HRMS (ESI^+^) [M + H]^+^ calcd for C_35_H_46_N_9_O_3_F_2_ 678.3692 found 678.3685.

##### 6-Guanidino-*N*-(4-(3-guanidinopropoxy)-2-(4-phenylpiperidine-1-carbonyl)phenyl)hexanamide dihydrochloride (8c)

The diguanidinium compound **8c** was prepared from the diamine **7c** according to the method used to prepare **8a**. The product **8c** was obtained as a semiglassy solid (45 mg, 56%); δ_H_ (methanol-*d*_4_) 1.47 (m, 2H), 1.62 (m, 2H), 1.73 (methylene envelope, 4H), 1.96 (m, 1H), 2.07 (m, 2H), 2.40 (t, *J *= 7.2 Hz, 2H), 2.83–2.91 (complex, 2H), 3.19 (t, *J *= 6.8 Hz, 2H), 3.41 (t, *J *= 6.8 Hz, 2H), 3.49 (m, 1H), 3.75 (br d, *J*=12.8 Hz, 1H), 4.11 (t, *J *= 5.6 Hz, 2H), 4.73 (br d, *J *= 11.6 Hz, 1H), 6.94 (s, 1H), 7.05 (dd, *J *= 2.8, 8.8 Hz, 1H), 7.16–7.33 (complex, 6H); δ_C_ (methanol-*d*_4_) 26.3, 27.1, 29.54, 29.58, 33.9, 34.6, 36.7, 39.4, 42.2, 43.5, 66.4, 114.0, 116.9, 127.4, 127.7, 128.3, 129.0, 129.4, 134.1, 146.6, 158.0, 158.5, 158.6, 169.8, 174.9; *m/z* (ESI^+^, 50 eV): *m/z* (%): 551.5 (30) [M + H]^+^ HRMS (ES^+^) [M + H]^+^ calcd for C_29_H_43_N_8_O_3_ 551.3458 found 551.3431.

##### *N*-(2-(4-(Benzo[*d*][1,3]dioxol-5-yl)methyl)piperazine-1-carbonyl)-4-(3-guanidinopropoxy)phenyl-6-guanidinohexanamide dihydrochloride (8d)

The diguanidinium compound **8d** was prepared from the diamine **7d** according to the method used to prepare **8a**. The product **8d** was obtained as a glassy semisolid (50 mg, 93%); δ_H_ (methanol-*d*_4_) 1.40–1.55 (methylene envelope, 2H), 1.55–1.74 (methylene envelope, 4H), 2.06 (m, 2H), 2.35 (t, *J *= 7.2 Hz, 2H), 2.43 (br s, 2H), 2.53 (br s, 2H), 3.17–3.26 (br t, *J *= 6.7 Hz, 2H), 3.34–3.43 (complex, 4H), 3.50 (s, 2H), 3.71 (br s, 2H), 4.08 (m, 2H), 5.92 (s, 2H), 6.76 (br s, 2H), 6.86 (br s, 1H), 6.88 (d, *J *= 2.7 Hz, 1H), 7.04 (dd, *J *= 2.6, 8.8 Hz, 1H), 7.28 (d, *J *= 8.8 Hz, 1H); δ_C_ (methanol-*d*_4_) 26.4, 27.2, 29.6, 36.9, 39.6, 42.4, 53.2, 53.4, 63.1, 66.6, 102.5, 109.0, 110.9, 114.3, 117.3, 124.3, 128.5, 129.0, 131.0, 133.6, 148.7, 149.3, 158.1, 170.0, 174.9; *m/z* (ESI^+^, 50 eV): *m/z* (%): 610.5 (20) [M + H]^+^. HRMS (ES^+^) [M + H]^+^ calcd for C_30_H_44_N_9_O_5_ 610.34 65 found 610.3474.

### 3.2. Biology

#### 3.2.1. Ca_v_2.2 Radioligand Displacement Assay

Radioligand binding assays were run in triplicate in 96-well plates at room temperature as previously described [39]. Each assay contained the test compound, radiolabelled peptide (7 pM ^125^I-GVIA) and 8 μg of crude rat brain membrane, added last. All dilutions were made in assay buffer (20 mM HEPES, 75 mM NaCl, 0.2 mM EDTA, 0.2 mM EGTA, 2 μM Leupeptin, 2 μL apoprotinin (to 30 mL assay buffer) and 0.1% BSA, pH 7.4). The final volume in each well was 150 μL. After shaking for 1 h, the membrane was filtered (Wallac, Finland glass fibre filters pre-soaked in 0.6% polyethyleneimine) and washed with 20 mM HEPES, 125 mM NaCl, pH 7.4 on a Tomtec harvester. After addition of scintillant, radioactivity bound to the filter was counted using a 1450 MicroBeta (Wallac, Finland). The data was analyzed using GraphPad Prism 2.0 (GraphPad Software, Inc, San Diego, USA).

#### 3.2.2. Fluorescence Measurement of Calcium Responses

SH-SY5Y cells were plated at a density of 30,000 cells/well on 384-well black-walled imaging plates and loaded for 30 min at 37 °C with Calcium 4 no-wash dye (Molecular Devices, Sunnyvale, CA) diluted in physiological salt solution (PSS; composition: 140 mM NaCl, 11.5 mM glucose, 5.9 mM KCl, 1.4 mM MgCl_2_, 1.2 mM NaH_2_PO_4_, 5 mM NaHCO_3_, 1.8 mM CaCl_2_, 10 mM 4-(2-hydroxyethyl)-1-piperazineethanesulfonic acid (HEPES), pH 7.4). SH-SY5Y cells represent an attractive model system and express human Ca_v_2.2 channels in a physiologically relevant context since in native, systems Ca_v_ channels are most likely co-expressed with auxiliary α2δ and β subunits.

Calcium responses, elicited by addition of 90 mM KCl and 5 mM CaCl_2_ in the presence of 10 µM nifedipine, were measured using a FLIPR^TETRA^ fluorescent plate reader (excitation, 470–495 nm; emission, 515–575 nm) after 5 min pre-treatment with test compounds in PSS containing 1.8 mM CaCl_2_. Under these conditions, the Ca^2+^ response elicited by addition of KCl/CaCl_2_ is mediated predominantly by GVIA-sensitive Ca_v_2.2 [43]. ω-Conotoxin CVID (3 µM) was included as a positive control to define maximal inhibition of Ca_v_2.2 responses.

Fluorescent responses were plotted as response over baseline using ScreenWorks (Molecular Devices, version 3.1.1.4). Concentration-response curves of peak calcium responses, normalized to control responses, were generated using GraphPad Prism (Version 4.00, San Diego, California) using a 4-parameter Hill equation with variable Hill slope fitted to the data.

#### 3.2.3. Patch Clamp Electrophysiology

HEK293 cells stably expressing human Ca_v_2.2 (*α1B* + *α_2_ δ* + *β_3_*), were plated onto 12 mm coverslips and used for whole-cell patch clamp experiments [47]. Depolarization-activated calcium currents were obtained with the whole-cell recording configuration at room temperature (23–25 °C), using a MultiClamp 700B amplifier and pClamp9.2 software (Molecular Devices, Sunnyvale, CA). The external bath solution contained (in mM) 90 NaCl; 10 CaCl_2_; 1 MgCl_2_; 10 HEPES; 30 TEA-Cl; 5 CsCl; 10 glucose, pH 7.4 with TEA-OH (~310 mOsmol/kg). The patch pipette had a resistance of 1–3 MΩ, with an internal solution composed of (in mM) 130 K-Gluconate; 2 MgCl_2_; 5 EGTA; 10 HEPES; 5 NaCl; 2 Mg-ATP; 1 Li-GTP, pH 7.2 with Cs-OH (~300 mOsmol/kg). Calcium currents were elicited from a holding potential of −80 mV with a depolarizing voltage step to a test potential of +20 mV for 150 ms, applied every 10 s. Bath perfusion was used to apply compounds, with series resistance typically compensated at 60%−80% and leak subtraction using a −P/4 pulse protocol. 

## 4. Conclusions

The diphenylmethylpiperazine moiety is less likely to lead to toxic effects than the phenoxyaniline substituent present in previously investigated anthranilamide-based GVIA mimics: Anilides are listed in structural alerts for potential adverse drug effects [48] with hepatotoxicity of anilides in certain cases being linked to P-450 activation to benzoquinoneimines in humans [49]. It was therefore pleasing to see that mimics bearing the diphenylmethylpiperazine pharmacophore (**7a**, **7b**, **8a**, **8b**) showed very similar activities in the radioligand displacement assay, and improved inhibition of intracellular calcium responses in SH-SY5Y cells, relative to the best of the phenoxyaniline-bearing compounds. Two sets of related compounds that did not possess the diphenylmethyl substituent (**7c**, **7d**, **8c**, **8d**) were inactive in the radioligand displacement assay, further suggesting that the diphenylmethyl moiety enhances calcium channel affinity in this class of compound.

The radioligand displacement assay employing ^125^I-ω-conotoxin GVIA is thought to be a specific measure of binding affinity to Ca_v_2.2 channels, because ω-conotoxin GVIA is exceptionally selective for this channel. As this assay does not measure the actual passage of Ca^2+^ ions though the channel, there is always the possibility that test compounds could cause the ^125^I-ω-conotoxin to be released from the channel through, for example, an allosteric effect, without actually inhibiting the channel. It was therefore also very pleasing to find that ω-conotoxin GVIA mimetics (**7c**, **7d**, **8c**, **8d**) identified to be active in the radioligand displacement assay also induced functional inhibition of intracellular calcium responses in SH-SY5Y neuroblastoma cells and calcium currents in HEK293 cells stably expressing human Ca_v_2.2 channels. These data fill an important gap between binding and downstream functional inhibition on median nerve-stimulated rat vas deferans that we have previously observed [50]. These results therefore validate both the use of ω-conotoxin GVIA as a starting point for the design of Ca_v_2.2 channel blockers and the use of the radioligand displacement assay as an initial screening tool for Ca_v_2.2 activity. 

The results from the functional assays show the difluoro, diguanidino compound **8b** to be the most effective Ca_v_2.2 channel blocker of the compounds examined in this study. The results obtained with this compound will form the basis for further optimization work, with the aim of improving potency and incorporating more drug-like properties.
